# Clinical Characteristics of COVID-19 Patients Infected by the Omicron Variant of SARS-CoV-2

**DOI:** 10.3389/fmed.2022.912367

**Published:** 2022-05-09

**Authors:** Jianguo Zhang, Nan Chen, Daguo Zhao, Jinhui Zhang, Zhenkui Hu, Zhimin Tao

**Affiliations:** ^1^Department of Emergency Medicine, The Affiliated Hospital, Jiangsu University, Zhenjiang, China; ^2^Jiangsu Province Key Laboratory of Medical Science and Laboratory Medicine, School of Medicine, Jiangsu University, Zhenjiang, China; ^3^Department of Critical Care Medicine, The First Affiliated Hospital of Soochow University, Suzhou, China; ^4^Department of Critical Care Medicine, The Affiliated Hospital, Jiangsu University, Zhenjiang, China

**Keywords:** SARS-CoV-2, omicron variant, COVID-19, pathogenicity, vaccination

## Abstract

**Background:**

Currently, as the omicron variant of severe acute respiratory syndrome coronavirus 2 (SARS-CoV-2) surges amid the coronavirus disease 2019 (COVID-19) pandemic, its clinical characteristics with intrinsic severity and the protection from vaccination have been understudied.

**Methods:**

We reported 169 COVID-19 patients that were infected with the omicron variant of SARS-CoV-2 and hospitalized in Suzhou, China, from February to March 2022, with their demographic information, medical/immunization history, clinical symptom, and hematological profile. At the same time, patients with none/partial (one-dose), full (two-dose) and three–dose vaccination were also compared to assess the vaccine effectiveness.

**Findings:**

For the omicron COVID-19 patients included in this study, their median age was 33.0 [interquartile range (IQR): 24.0–45.5], 53.3% were male and the median duration from illness onset to hospitalization was 2 days. Hypertension, bronchitis, and diabetes were the leading comorbidities among patients. While the common clinical symptoms included cough, fever, expectoration, and fatigue, etc., asymptomatic patients took up a significant portion (46.7%). For hematological parameters, most values revealed the alleviated pathogenicity induced by the omicron variant infection. No critically ill or deceased patients due to COVID-19 infection were reported in this study.

**Interpretation:**

Our results supported that the viremic effect of the omicron variant became milder than the previous circulating variants, while full vaccination or booster shot was greatly desired for an effective protection against clinical severity.

## Introduction

The unprecedented pandemic of coronavirus disease 2019 (COVID-19) started more than 2 years ago (1). Ever since, the world has been jolted by serial waves of COVID-19 outbreaks triggered by the evolving mutants from the responsible pathogen, i.e., severe acute respiratory syndrome coronavirus 2 (SARS-CoV-2) (2, 3). So far, the alpha, beta, gamma, delta, and omicron variants of SARS-CoV-2 have been designated as variants of concern (VOCs) with high infectivity and virulence, while each later one surfaces with the higher transmissibility than the previous (4). As of March 20, 2022, the reported COVID-19 cases exceeded 468 million with an estimated fatality rate of 1.3% (5).

Presently, the omicron variant of SARS-CoV-2 outpaces others to be the dominant circulating strain, sweeping across the world (6). The major omicron sublineages that prevail among the local COVID-19 outbreaks in China are BA.1 and BA.2 (7–9). It was first discovered in November 2021 in South Africa, when the early study on the characteristics and outcomes of hospitalized COVID-19 patients infected by the omicron variant indicated that the infection was associated with significantly lessened length of hospital stays and reduced severity and mortality, when compared to the previous COVID-19 hits (10, 11). However, the omicron variant possessed much more mutations in viral genome than any of the other VOCs (12). Furthermore, convalescent sera from recovered patients infected by the alpha, beta or delta variant could not neutralize the omicron variant, while sera from fully vaccinated persons (two doses of mRNA or vector vaccines) enabled neutralization of the omicron variant to a lesser extent than that of the delta variant (13). For those reasons, there are raising concerns about whether the immune evasion and pathogenic influence of the omicron variant would be more severe than the previous strains.

In the earlier reports we analyzed and compared the clinical characteristics between patients infected by the wild-type or delta variant SARS-CoV-2 (14, 15). Herein we investigated the demographic information and baseline characteristics of confirmed COVID-19 patients infected with the omicron variants during the recent coronavirus flareup in the city of Suzhou, China, in February and March 2022. Through this study we seek to understand the clinical manifestations of COVID-19 patients infected by the omicron variant of SARS-CoV-2 and how the vaccination status might protect from severity.

## Methods

### Patient Information

The retrospective study included 169 COVID-19 patients who were admitted to the Fifth People's Hospital of Suzhou (TFPHS, the Affiliated Infectious Diseases Hospital of Soochow University), Jiangsu Province, China, from February 13 to March 21, 2022. COVID-19 infections were confirmed as reported (16). Exclusion criteria were as follows: patients with malignancy, pregnancy, blood disease, or autoimmune deficiency, and patients who failed to complete blood examinations, and patients who were younger than 12 years. The study was approved by the Research Ethics Commission of TFPHS. Patient information remained anonymous, and written consents were waived due to a major infectious disease outbreak.

### Procedure and Vaccination

COVID-19 patients infected by the omicron variant of SARS-CoV-2 were hospitalized and treated as reported (17). Blood cell analysis was conducted by an automated XN1000 hematology analyzer (SYSMEX, Japan), and biochemical indicators were analyzed using VITROS 350 autoanalyzer (Johnson &. Johnson, USA). Computed tomography (CT) was performed using BrightSpeed 16 CT Scanner (GE Healthcare, USA). The scanning parameters were set as 120 kVp, 80 mA, 1.5-mm collimation, reconstruction matrix of 512 × 512, slice thickness of 5.0 mm, scan field of view (FOV) of 25 × 25 cm, and high spatial resolution algorithm. For most of admitted COVID-19 patients in TFPHS, two types of inactivated vaccines (Sinovac or Sinopharm) have been administered. Serological tests of patients based on detection of SARS-CoV-2-specific immunoglobulin M (IgM) and immunoglobulin G (IgG) were conducted, using 2019-nCoV Ab test kit (colloidal gold), manufactured by Innovita Biological Technology Co. Ltd., China.

### Statistical Analysis

Data were summarized as the median and IQR values for continuous variables and frequencies for categorical variables. For comparisons between two groups, Mann-Whitney U test was used for continuous variables. Categorical variables were examined by Chi-squared test. All calculated *p*-values were two-sided, and *p*-values < 0.05 were considered statistically significant. All statistical analyses were performed using SPSS version16.0 (SPSS Inc., Chicago, IL).

## Results

### Baseline Characteristics of COVID-19 Patients Infected by the Omicron Variant of SARS-CoV-2

In this study 169 COVID-19 patients infected by the omicron variant of SARS-CoV-2 were hospitalized in Suzhou, Jiangsu Province, China, from February to March 2022. Their median age was 33.0 (IQR: 24.0–45.5), 53.3% were male, and the median duration time from illness onset to hospitalization was 2.00 days (IQR: 2.00–3.00) (Table 1). We further grouped the patients into three subgroups; that is, one with none (34 patients, 20.1%) or partial (one-dose) vaccination (12 patients, 7.1%) (a total of 46 patients or 27.2% of the total patients in this subgroup), one with full (two-dose) vaccination (78 patients, 46.2%), and one that received booster shots (i.e., three-dose vaccination) (45 patients, 26.6%). Then, demographic information, medical history, clinical symptom, and antibody response were analyzed for all patients, together with comparisons of those baseline characteristics between patients none/partially vaccinated and patients fully vaccinated (indicated by *p*^1^ values), and between patients none/partially vaccinated and patients three doses vaccinated (indicated by *p*^2^ values) (Table 1).

**Table 1:** Demographic information, medical/immunization history, clinical symptom, and antibody production in the COVID-19 patients infected by the omicron variant in Suzhou, China, in February and March 2022.

	**Total** **(*n* = 169)**	**None or partially vaccinated** **(*n* = 46)**	**Fully** **vaccinated (*n* = 78)**	***p^**1**^***	**Three doses** **vaccinated (*n* = 45)**	***p^**2**^***
Age (year)	33.0 (24.0–45.5)	32.5 (23.0–58.5)	31.0 (21.0–47.0)	0.380	36.0 (28.50–41.0)	0.668
Gender, male (%)	90 (53.3)	24 (52.2)	40 (51.3)	0.924	26 (57.8)	0.591
Onset to hospitalization, day	2.00 (2.00–3.00)	2.00 (2.00–3.00)	2.00 (1.00–2.00)	0.057	2.00 (2.00–3.00)	0.414
**Comorbidity (%)**						
Hypertension	19 (11.2)	9 (19.6)	7 (9.0)	0.089	3 (6.7)	0.069
Bronchitis	4 (2.4)	3 (6.5)	1 (1.3)	0.285	0 (0)	0.248
Diabetes	3 (1.8)	1 (2.2)	1 (1.3)	1.000	1 (2.2)	1.000
**Symptoms**						
Asymptomatic	79 (46.7)	22 (47.8)	42 (53.8)	0.517	15 (33.3)	0.159
Cough	56 (33.1)	13 (28.3)	25 (32.1)	0.658	18 (40)	0.237
Fever	51 (30.2)	18 (39.1)	22 (28.2)	0.209	11 (24.4)	0.133
Sore throat	24 (14.2)	2 (4.3)	8 (10.2)	0.409	14 (31.1)	0.001
Expectoration	20 (11.8)	6 (13.0)	6 (7.7)	0.510	8 (17.8)	0.531
Fatigue	15 (8.9)	6 (13.0)	5 (6.4)	0.353	4 (8.9)	0.765
Diarrhea	3 (1.8)	1 (2.2)	1 (1.3)	1.000	1 (2.2)	1.000
Vomiting	2 (1.2)	2 (4.3)	0 (0)	0.136	0 (0)	0.495
Abdominal pain	1 (0.6)	1 (2.2)	0 (0)	0.371	0 (0)	1.000
**Antibody production (%)**						
None	61 (36.1)	30 (65.2)	31 (39.7)	0.006	0 (0)	<0.001
Only IgG	106 (62.7)	16 (34.8)	45 (57.7)	0.014	45 (100)	<0.001
Only IgM	0 (0)	0 (0)	0 (0)	–	0 (0)	–
IgG + IgM	2 (1.2)	0 (0)	2 (2.6)	0.530	0 (0)	–

*Comparisons were performed between patients none/partially vaccinated and patients fully vaccinated (exhibited by p^1^ values) or patients who were three doses vaccinated (exhibited by p^2^ values)*.

Among all patients, hypertension, bronchitis, and diabetes were the leading comorbidities. Notably, in addition to those with typical symptoms of cough, fever, sore throat, expectoration, and fatigue, etc., asymptomatic patients occupied a nearly half portion of total infections. Irrespective of immunization status, 36.1% COVID-19 patients infected by the omicron variant did not develop antibody response, while 62.7% produced only IgG and only 1.2% produced both IgG and IgM. There was no noticeable difference between patients fully vaccinated or booster shot (three doses) vaccinated and patients none/partially vaccinated in terms of the baseline characteristics, except that IgG production significantly increased as the vaccination times added up.

### Laboratory Parameters of COVID-19 Patients Infected by the Omicron Variant of SARS-CoV-2

A substantial portion of the omicron COVID-19 patients demonstrated abnormal levels of white blood cells, neutrophils, lymphocytes, and monocytes, showing signs of leukocytosis, neutrophilia, lymphocytopenia and monocytosis (Table 2). In contrast, the count of red blood cells (RBCs), and the levels of hemoglobin and hematocrit among most omicron COVID-19 patients remained within the normal range, indicating that anemia was insignificant among the majority of patients. Similarly, thrombocytopenia was also marginal with only 4.1% patients tested abnormal, as the platelet levels in most omicron variant infections were regular. Nevertheless, coagulopathy was found in a moderate proportion of omicron COVID-19 patients. For instance, the D-dimer levels of most patients fell in the normal range, still leaving 11.8% patients (20 out of 169) with abnormally high values. Similar coagulopathic incidents included the prolonged prothrombin time and activated partial thromboplastin time. Thereby, examining the viremia of the omicron variant on blood profiles of patients, mild hematological impairment was spotted, implying a modest degree of virulence.

**Table 2:** Baseline characteristics of COVID-19 patients infected by the omicron variant in their hematological profiles.

	**Normal range**	**Total (*n* = 169)**	**Abnormal values**	**Patients with abnormal values**
**Blood cell count**				
White blood cells, × 10^9^/L	3.5–9.5	6.02 (5.14–7.41)	>9.5	16 (9.5)
Neutrophils, × 10^9^/L	1.8–6.3	4.22 (3.05–5.59)	>6.3	25 (14.8)
Lymphocytes, × 10^9^/L	1.1–3.2	1.07 (0.70–1.62)	<1.1	89 (52.7)
Monocytes, × 10^9^/L	0.1–0.6	0.57 (0.41–0.71)	>0.6	71 (42.0)
Red blood cells, × 10^12^/L	3.8–5.1	4.77 (4.50–5.25)	<3.8	1 (0.6)
Hemoglobin, g/L	115–150	139.00 (129.00–154.00)	<115	10 (5.9)
Hematocrit, %	35–50	41.80 (39.10–45.65)	<35	5 (3.0)
MCV, fL	82–100	88.00 (85.05–90.55)	<82	19 (11.2)
MCH, pg	27–34	29.40 (28.45–30.35)	<27	12 (7.1)
MCHC, gL	316–354	334.00 (327.00–341.00)	<316	12 (7.1)
RDW, %	11–16	12.00 (11.70–12.50)	>16	5 (3.0)
Platelet, × 10^9^/L	125–350	221.00 (182.00–261.00)	<125	7 (4.1)
MPV, fL	7.4–12.5	9.70 (9.05–10.50)	>12.5	2 (1.2)
PDW, %	9–17	12.80 (10.45–16.00)	>17	9 (5.3)
**Coagulation factors**				
Prothrombin time, s	9–13	11.50 (10.55–12.65)	>13	24 (14.2)
INR	0.8–1.2	0.95 (0.88–0.99)	>1.2	2 (1.2)
aPTT, s	23.3–32.5	30.00 (26.65–33.95)	>32.5	59 (34.9)
Thrombin time, s	14–21	18.40 (15.25–19.30)	>21	2 (1.2)
Fibrinogen, g/L	2–4	2.75 (2.27–3.21)	>4	6 (3.6)
D–dimer, mg/L	<0.55	0.23 (0.15–0.38)	>0.55	20 (11.8)
**Metabolic & biomarker panel**				
CRP, mg/L	0–10	5.00 (2.18–9.90)	>10	35 (20.7)
Procalcitonin, ng/mL	<0.1	0.15 (0.08–0.21)	>0.1	105 (62.1)
Total bilirubin, μmol/L	3–22	7.60 (5.60–11.20)	>22	3 (1.8)
Direct bilirubin, μmol/L	0–5	2.40 (1.20–3.40)	>5	9 (5.3)
Indirect bilirubin, μmol/L	0–19	5.50 (3.45–8.20)	>19	5 (3.0)
ALT, U/L	9–50	31.00 (25.00–45.00)	>50	23 (13.6)
AST, U/L	15–40	25.00 (20.50–32.00)	>40	19 (11.2)
ALP, U/L	32–126	73.00 (58.50–99.50)	>126	29 (17.2)
GGT, U/L	12–73	20.00 (14.00–30.50)	>73	6 (3.6)
Total protein, g/L	63–82	77.70 (72.95–82.10)	<63	2 (1.2)
Albumin, g/L	35–50	44.90 (42.50–47.60)	<35	2 (1.2)
Globulin, g/L	20–30	32.00 (27.85–38.10)	<20	1 (0.6)
BUN, mmol/L	2.86–8.2	4.43 (3.58–5.32)	>8.2	3 (1.8)
Creatinine, mmol/L	31.7–133	58.60 (41.63–71.00)	>133	2 (1.2)
LDH, U/L	80–285	218.00 (177.50–350.50)	>285	64 (37.9)
CPK, U/L	38–174	64.00 (45.00–97.00)	>174	12 (7.1)
Glucose, mmol/L	3.89–6.11	6.10 (5.55–6.85)	>6.11	84 (49.7)
Cholesterol, mmol/L	2.3–5.2	4.63 (3.96–5.33)	>5.2	50 (29.6)
Triglyceride, mmol/L	0.4–1.7	0.92 (0.61–1.34)	>1.7	25 (14.8)
Potassium, mmol/L	3.5–5.3	4.05 (3.84–4.24)	<3.5	6 (3.6)
Sodium, mmol/L	137–147	138.45 (135.33–140.38)	<137	67 (39.6)
Total calcium, mmol/L	2.1–2.55	2.33 (2.26–2.40)	<2.1	8 (4.7)

*For each parameter, the patient number (N) and proportion (%) with abnormal values were calculated and indicated as N (%). MCV, mean corpuscular volume; MCH, mean corpuscular hemoglobin; MCHC, mean corpuscular hemoglobin concentration; RDW, red blood cell distribution width; MPV, mean platelet volume; PDW, platelet distribution width; aPTT, activated partial thromboplastin time; INR, international normalized ratio; CRP, c-reaction protein; ALT, alanine aminotransferase; AST, aspartate aminotransferase; ALP, alkaline phosphatase; GGT, γ-glutamyl transferase; BUN, blood urea nitrogen; LDH, lactic dehydrogenase; CPK, creatine phosphokinase*.

Most biochemical indicators in the omicron COVID-19 patients revealed the mild impact. Markedly, the median level of procalcitonin in all patients was abnormally elevated with 62.1% patients possessing higher values than normal. Similarly, the portions of patients with aberrant values of c-reactive proteins, alanine aminotransferase, aspartate aminotransferase, alkaline phosphatase, lactic dehydrogenase, glucose, cholesterol, triglyceride, and sodium were substantial or considerable. Those results indicated that the infection of the omicron variant still caused noticeable injuries on major organs, such as liver and heart. As shown in Table 3, compared to patients who were none/partially vaccinated, patients fully vaccinated did not exhibit a significant difference in their hematological profile, and patients with booster vaccination demonstrated some alleviated characteristics, including mitigations in thrombocytopenia, thrombin time prolonging, and alkaline phosphatase elevation, with most baseline characteristics undifferentiable from those in patients who were none/partially vaccinated.

**Table 3:** The hematological profiles of COVID-19 patients infected by the omicron variant were divided into three subgroups and thereby compared between patients with none or partial vaccination and patients with full vaccination (exhibited by *p*^1^ values), or between patients with none or partial vaccination and patients with three-dose vaccination (exhibited by *p*^2^ values).

	**Normal range**	**None or partially vaccinated (*n* = 46)**	**Fully vaccinated** **(*n* = 78)**	***p^**1**^***	**Three doses vaccinated (*n* = 45)**	***p^**2**^***
**Blood cell count**						
White blood cells, × 10^9^/L	3.5–9.5	6.39 (5.17–7.23)	5.86 (4.94–7.34)	0.799	6.49 (5.27–8.04)	0.480
Neutrophils, × 10^9^/L	1.8–6.3	4.24 (2.71–5.48)	4.13 (2.86–5.44)	0.877	4.39 (3.34–6.01)	0.414
Lymphocytes, × 10^9^/L	1.1–3.2	0.82 (0.60–1.50)	1.09 (0.77–1.60)	0.161	1.20 (0.83–1.77)	0.104
Monocytes, × 10^9^/L	0.1–0.6	0.61 (0.38–0.74)	0.54 (0.04–0.69)	0.474	0.57 (0.43–0.66)	0.754
Red blood cells, × 10^12^/L	3.8–5.1	4.69 (4.36–5.17)	4.78 (4.52–5.17)	0.364	5.00 (4.50–5.34)	0.121
Hemoglobin, g/L	115–150	137.50 (129.00–153.00)	137.00 (129.00–150.00)	0.715	146.00 (131.00–158.00)	0.156
Hematocrit, %	35–50	41.10 (38.83–45.18)	41.25 (38.70–44.35)	0.871	43.80 (39.70–47.25)	0.084
MCV, fL	82–100	87.75 (84.35–90.68)	87.55 (84.58–90.20)	0.729	89.00 (85.70–90.85)	0.482
MCH, pg	27–34	29.30 (28.70–30.70)	29.20 (28.30–30.30)	0.302	29.90 (28.75–30.35)	0.625
MCHC, gL	316–354	336.50 (328.50–342.00)	333.00 (327.00–339.00)	0.276	335.00 (326.50–343.50)	0.812
RDW, %	11.5–17.8	12.10 (11.90–12.35)	12.00 (11.60–12.55)	0.531	12.00 (11.65–12.50)	0.292
Platelet, × 10^9^/L	125–350	200.50 (167.00–251.75)	221.00 (180.00–262.25)	0.129	236.00 (197.00–269.00)	0.022
MPV, fL	7.4–12.5	9.75 (9.08–10.70)	9.70 (8.90–10.55)	0.744	9.90 (9.20–10.40)	0.769
PDW, %	9–17	13.10 (10.38–15.93)	13.10 (10.38–16.00)	0.840	12.10 (11.00–16.05)	0.691
**Coagulation factors**						
Prothrombin time, s	9–13	11.50 (10.58–12.83)	11.40 (10.58–12.43)	0.621	11.50 (10.50–12.65)	0.656
INR	0.8–1.2	0.97 (0.90–1.00)	0.95 (0.88–0.99)	0.367	0.95 (0.88–0.99)	0.192
aPTT, s	23.3–32.5	29.85 (26.55–35.63)	30.15 (27.40–33.98)	0.924	28.70 (25.50–33.90)	0.272
Thrombin time, s	14–21	18.95 (15.30–19.60)	18.45 (15.45–19.20)	0.306	17.90 (14.90–19.00)	0.018
Fibrinogen, g/L	2–4	2.66 (2.25–3.16)	2.70 (2.10–2.96)	0.666	2.99 (2.54–3.41)	0.067
D–dimer, mg/L	<0.55	0.26 (0.16–0.47)	0.19 (0.15–0.36)	0.140	0.23 (0.15–0.37)	0.377
**Metabolic and biomarker panel**						
CRP, mg/L	0–10	4.47 (1.98–9.35)	5.50 (2.02–10.64)	0.459	4.90 (2.75–8.25)	0.779
Procalcitonin, ng/mL	<0.1	0.14 (0.08–0.19)	0.17 (0.09–0.21)	0.214	0.14 (0.08–0.21)	0.535
Total bilirubin, μmol/L	3–22	8.10 (5.60–11.18)	7.40 (5.50–12.05)	0.784	7.90 (6.10–10.85)	0.830
Direct bilirubin, μmol/L	0–5	2.40 (1.30–3.90)	2.30 (1.20–3.10)	0.269	2.40 (0.65–3.45)	0.376
Indirect bilirubin, μmol/L	0–19	5.55 (3.65–7.95)	5.00 (3.20–9.05)	0.959	5.60 (3.65–7.95)	0.886
ALT, U/L	9–50	30.00 (26.00–39.25)	33.00 (24.00–40.25)	0.891	31.00 (25.00–45.50)	0.573
AST, U/L	15–40	26 0.00 (21.75–35.75)	25.00 (20.75–32.00)	0.233	24.00 (20.00–28.00)	0.097
ALP, U/L	32–126	80.50 (58.75–121.00)	72.50 (60.00–103.50)	0.420	70.00 (57.00–82.00)	0.049
GGT, U/L	12–73	20.00 (13.75–30.25)	18.00 (14.00–26.25)	0.447	22.00 (15.00–45.00)	0.253
Total protein, g/L	63–82	77.55 (72.48–82.38)	77.45 (73.15–82.25)	0.844	78.80 (73.20–82.20)	0.827
Albumin, g/L	35–50	45.15 (42.08–47.33)	45.20 (42.73–47.90)	0.614	43.90 (42.55–47.45)	0.886
Globulin, g/L	20–30	31.35 (25.88–40.08)	31.35 (27.75–37.45)	0.992	33.20 (28.50–38.15)	0.578
BUN, mmol/L	2.86–8.2	4.19 (3.69–5.58)	4.50 (3.70–5.30)	0.899	4.32 (3.45–5.08)	0.815
Creatinine, mmol/L	31.7–133	59.00 (39.85–71.50)	54.35 (40.78–68.33)	0.534	63.20 (46.60–72.90)	0.453
LDH, U/L	80–285	219.50 (186.00–370.50)	219.50 (183.25–350.25)	0.603	204.00 (165.00–345.50)	0.184
CPK, U/L	38–174	66.50 (50.00–112.50)	64.00 (45.50–100.00)	0.358	62.00 (42.00–83.50)	0.081
Glucose, mmol/L	3.89–6.11	6.35 (5.85–6.73)	6.00 (5.40–6.80)	0.177	6.30 (5.65–7.11)	0.805
Cholesterol, mmol/L	2.3–5.2	4.34 (3.73–5.11)	4.62 (3.93–5.30)	0.179	4.92 (4.28–5.66)	0.007
Triglyceride, mmol/L	0.4–1.7	0.87 (0.59–1.30)	0.89 (0.58–1.24)	0.961	0.94 (0.66–1.53)	0.515
Potassium, mmol/L	3.5–5.3	3.97 (3.74–4.18)	4.06 (3.84–4.25)	0.178	4.09 (3.91–4.34)	0.063
Sodium, mmol/L	137–147	138.45 (135.25–139.93)	138.70 (135.45–141.10)	0.444	138.20 (135.25–140.20)	0.883
Total calcium, mmol/L	2.1–2.55	2.30 (2.22–2.38)	2.32 (2.26–2.43)	0.237	2.35 (2.27–2.41)	0.118

*MCV, mean corpuscular volume; MCH, mean corpuscular hemoglobin; MCHC, mean corpuscular hemoglobin concentration; RDW, red blood cell distribution width; MPV, mean platelet volume; PDW, platelet distribution width; aPTT, activated partial thromboplastin time; INR, international normalized ratio; CRP, c-reaction protein; ALT, alanine aminotransferase; AST, aspartate aminotransferase; ALP, alkaline phosphatase; GGT, γ-glutamyl transferase; BUN, blood urea nitrogen; LDH, lactic dehydrogenase; CPK, creatine phosphokinase*.

### CT Features of COVID-19 Patients Infected by the Omicron Variant of SARS-CoV-2

**Figure 1:**
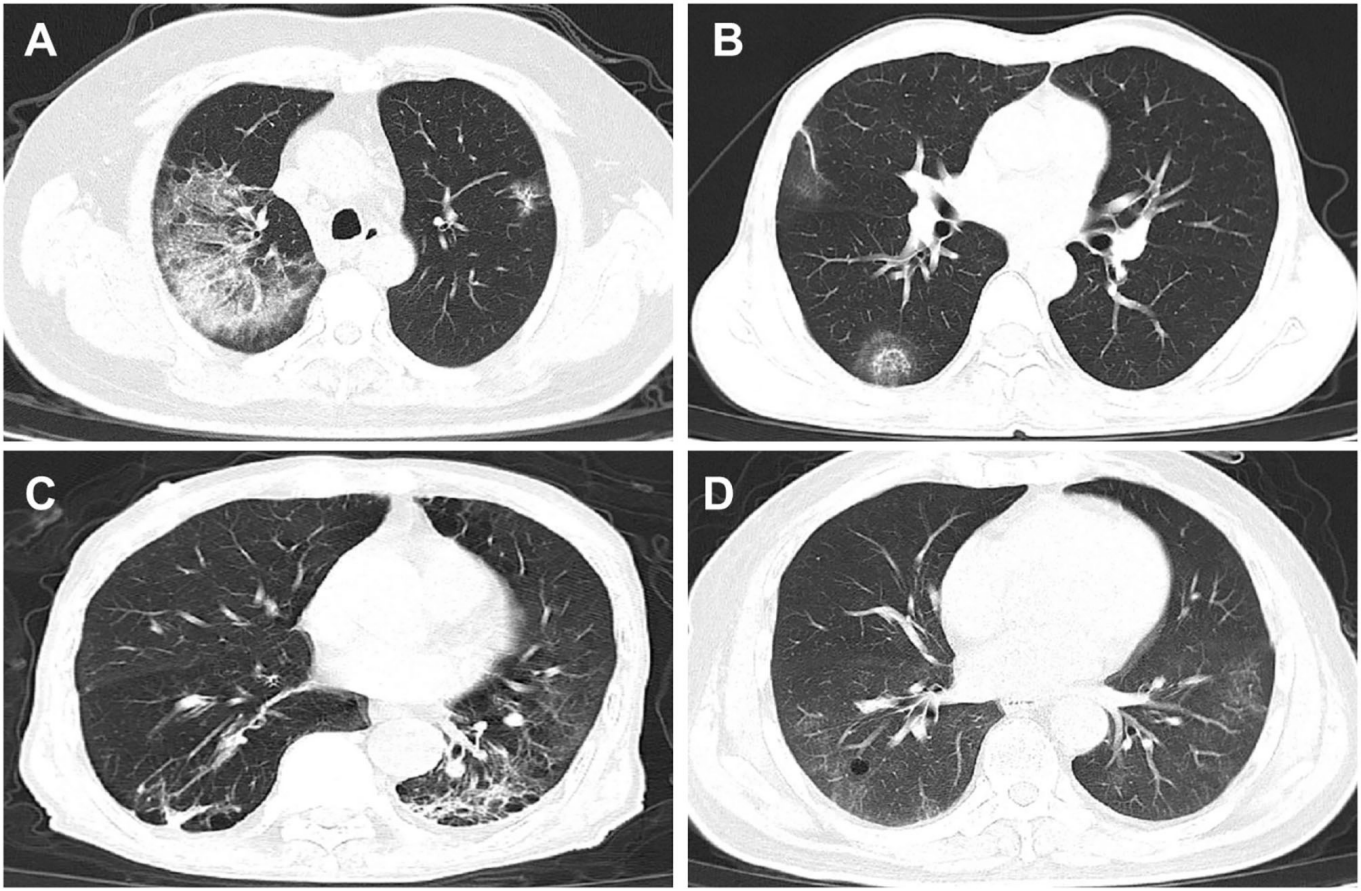
Selected CT graphs of COVID-19 patients infected by the omicron variant of SARS-CoV-2 in Suzhou, China in 2022, taken upon hospital admission, showing representative pathological changes in lungs. **(A)** From a 64-year-old man with fever and cough symptoms. Axial CT image showed GGOs and consolidation in the right upper lobe, taken on the fifth day from illness onset. **(B)** From a 53-year-old man having cough and fever. CT image showed rounded opacities in the right lower lobe, taken on the eighth day from illness onset. Lesions were peripherally distributed. **(C)** From a 61-year-old man with fever. CT image showed linear opacities in the right and left lobes, and lesions were peripherally distributed. Image was taken on the fifth day from illness onset. **(D)** From a 75-year-old man with cough and fever. Axial CT image showed GGOs and cavitation in the right lobe, and lesion distributions were central and peripheral. Image was taken on the tenth day from illness onset.

Table 4 lists all common CT features of the omicron COVID-19 patients in our study. The individual proportion of patients with each CT feature was calculated in each subgroup and compared between different subgroups. The pathological characters in patients' lungs exhibited a high occurrence of unilateral and bilateral involvement, lesions located at left or right lower lobes and peripheral distribution. CT features were typified by ground glass opacities (GGOs), linear opacities, and air bronchogram (Figure 1). Among all patients, the incidences of consolidation or craze paving pattern became much lessened, showing milder pathological changes in lungs caused by the omicron variant. Furthermore, compared to those in the patient subgroup of none/partial vaccination, the CT characteristics in the patient subgroup of full vaccination did not reveal any noticeable difference, while some CT features in the patient subgroup of booster vaccination, including the bilateral involvement, lesion location at right middle and lower lobes, and crazy paving pattern, showed much reduced incidence.

**Table 4:** The CT features of COVID-19 patients infected by the omicron variant were divided into three subgroups as indicated.

**CT feature**	**In total (%)**	**None or partially vaccinated (%)**	**Fully vaccinated (%)**	***p^**1**^***	**Three doses vaccinated (%)**	***p^**2**^***
**Lung involvement**						
Unilateral	30.8	21.7	32.1	0.218	37.8	0.094
Bilateral	46.7	58.7	46.2	0.177	35.6	0.027
**Location of lesions**						
Left upper lobe	22.5	26.1	24.4	0.830	15.6	0.217
Left lower lobe	45.6	47.8	44.9	0.750	44.4	0.746
Right upper lobe	20.1	26.1	20.5	0.473	13.3	0.127
Right middle lobe	31.4	37.0	35.9	0.906	17.8	0.040
Right lower lobe	57.4	69.6	56.4	0.146	46.7	0.027
**Predominant distribution**						
Central	3.0	2.2	1.3	1.000	6.7	0.593
Peripheral	46.7	41.3	51.3	0.283	44.4	0.762
Central + Peripheral	27.8	37.0	25.6	0.183	22.2	0.124
**CT pattern**						
GGO	39.1	45.7	37.2	0.353	35.6	0.327
Consolidation	1.8	2.2	2.6	1.000	0.0	1.000
GGO + Consolidation	17.2	19.6	20.5	0.899	8.9	0.146
Crazy paving pattern	6.5	13.0	6.4	0.353	0.0	0.037
Linear opacities	53.3	50.0	50.0	1.000	62.2	0.240
Rounded opacities	8.3	6.5	10.3	0.704	6.7	1.000
Air bronchogram	12.4	15.2	12.8	0.708	8.9	0.354
Halo sign	1.8	0.0	3.8	0.458	0.0	–
Nodules	5.3	8.7	5.1	0.687	2.2	0.371
Tree-in-bud sign	3.0	6.5	0.0	0.093	4.4	1.000
Interlobular septal thickening	7.7	6.5	10.3	0.704	4.4	1.000
Bronchiolar wall thickening	8.9	6.5	5.1	1.000	17.8	0.100
Cavitation	1.8	0.0	2.6	0.530	2.2	0.495
Pleural effusion	9.5	8.7	10.3	1.000	8.9	1.000
Pericardial effusion	0.0	0.0	0.0	-	0.0	–

*The patient proportion with each specific CT feature was compared between none or partially vaccinated group and fully vaccinated group (exhibited by p^1^ values), or between none or partially vaccinated group and three doses vaccinated group (exhibited by p^2^ values). GGO, ground–glass opacity*.

## Discussion

Early studies reported by South African researchers, where the omicron variant was first discovered after nearly half population had been vaccinated and over half population had been exposed to SARS-CoV-2, suggested much attenuated pathogenicity with plummeted severity and mortality during the wide spreading of the omicron variant (10, 11, 18). Similar findings were also reported from other countries, including the United States, France, and South Korea (19–21), where vaccination coverage and population infection were both substantially high. Thereby, questions remain whether this reduced pathogenicity is due to the weakened intrinsic viremia or the strengthened acquired immunity by previous infection or/and sufficient vaccination, or both.

Differing from most of other countries, China has a high vaccination coverage but a low population of COVID-19 infection where reinfection cases are rare. Therefore, the acquired immunity against COVID-19 basically comes from effective vaccination rather than previous natural infection. Here our study that included 169 COVID-19 patients infected with the omicron variant of SARS-CoV-2 demonstrated a reduced clinical severity where mild infection profiles were observed. No critically ill or deceased patients were reported due to the omicron infection. This result mirrors an attenuated pathogenicity of the omicron variant compared to that induced by the wild-type strain or other VOCs and accents the importance of timely vaccination (with a booster shot) in order to significantly reduce the severity and lower the fatality.

Being a rapidly evolving RNA virus, SARS-CoV-2 recently mutates into its omicron variant with a much higher effective reproduction number than that of the delta variant (3.6–4.2 times), demonstrating an astounding infectivity and transmissibility (22, 23). Insofar, among all five VOCs, the omicron variant possessed the highest mutations in the genome structure (~50 mutations), where more than 32 mutations occurred in the spike protein (24). Those mutations take responsibility for the enhanced binding capacity to angiotensin-converting enzyme 2 (ACE2) (e.g., T478K, N501Y) and/or the increased cleavage activity by host furin (e.g., N679K, P681H), leading to much elevated infectivity and transmissibility of this variant; simultaneously, particular amino acid changes (e.g., E484A) in the spike protein enable to dodge the neutralizing antibodies, which eventually results in the heightened ability of immune escape (12, 15, 24, 25).

As a matter of fact, convalescent sera from the wild-type SARS-CoV-2 infection revealed a significantly lower degree of neutralization against the omicron variant than the delta variant (26). Sera from unvaccinated individuals infected with the alpha, beta, or delta variant of SARS-CoV-2 barely neutralized the omicron variant (13). Similarly, sera from patients infected by the omicron variant had residual cross-reactivity with other VOC (27). In parallel, sera from fully (two doses) vaccinated individuals reacted the least with the omicron variant among all reactions to VOCs (28). Those could explain why the breakthrough infection incidents in the omicron COVID-19 cases occurred frequently regardless of previous infection or vaccination history. Nevertheless, a booster vaccine, irrespective of vaccine type (e.g., mRNA or inactivated), could be efficient in improving the production of the neutralizing antibodies against the omicron variant infection, so offering effective protection from symptomatic infection or severe illness (26, 29–31). Notably, this neutralization response and vaccine effectiveness wane over time. Here our results came in line with those facts, showing that more than half proportion of patients with none or incomplete vaccination generated no antibody response. At the same time, among all patients infected by the omicron variant of SARS-CoV-2, antibody production increased as the dosing times of vaccines added.

Upon viral invasion, only a small subset of antibodies produced by B cells in the host is able to neutralize, while the majority of non-neutralizing antibodies as generated, albeit they do not counteract the viral infectivity, initiates the opsonophagocytic process by one region binding specifically to the viral particles via opsonization and the other region (Fc region) activating the Fc-receptor-mediated endocytosis of viral particles by phagocytes, such as natural killer cells, neutrophils, monocytes and macrophages (32). Since the non-neutralizing antibodies *per se* cannot nullify the viral infectivity, this antibody-dependent enhancement might be a double-bladed sword, mitigating or worsening the viral infection (32). Nevertheless, for a genetically labile RNA virus, such as influenza virus or human immunodeficiency virus (HIV), the non-neutralizing antibodies have been proven to contribute significantly to efficient viral clearance (33, 34). So far, those functional non-neutralizing antibody responses have been demonstrated to render protection against SARS-CoV-2 infection in its wild-type, alpha, beta, epsilon, and gamma form (35–37). Whether this protection reoccurs against other highly mutated SARS-CoV-2 variants, including delta and omicron, awaits to be soon unraveled. Our results showed that the omicron variant infection resulted in a substantial proportion of patients with signs of leukocytosis, neutrophilia, lymphocytopenia, monocytosis and coagulopathy, while leaving the levels and the major functional indices of RBCs and platelets minimally harmed. This corroborates the active interaction between the cell immunity and the omicron variant.

Beside the antibody-mediated immunity, the cell-mediated immunity induced by infection or vaccination has shown largely preserved T cell responses to the omicron variant (38–40). It has been hypothesized that memory CD4^+^ T cells mainly target the conserved motif in the spike protein that harbors a minority of mutations, where CD8^+^ T cells are frequently directed to the mutation site in the SARS-CoV-2 (38, 39). When encountering the omicron variant of SARS-CoV-2, memory CD4^+^ T cell responses wakened by previous infection or vaccination remain intact (41). On the other hand, only one low-prevalence epitope in the spike protein has been found to undertake single amino acid change (T95I) in the omicron variant, where CD8^+^ T cell recognition can be minimally compromised (42). Therefore, despite the fact that the omicron variant owns the highest mutations among the five VOCs, its T cell escape is minimal and comparable to other VOCs. On top of that, a booster vaccine effectively enhances T-cell responses (41, 43).

Due to key mutations in the spike protein of SARS-CoV-2 omicron variant, especially Q493R and N501Y, it binds to human and mouse ACE2 with much higher affinity than the wild type or other VOCs (44). However, viral entry into the host cells via ACE2 has to be primed and facilitated by transmembrane serine protease 2 (TMPRSS2), which is efficiently utilized by the wild type or the alpha, beta and delta variants, but not the omicron variant, possibly owing to the critical mutations at S1/S2 region and the reduced cleavage (45, 46). Thus, the omicron variant may enter the host via a differing endocytotic pathway from the wild type and other variants. As a result, the replication of the omicron variant is significantly attenuated, leading to mitigated pro-inflammatory responses, diminished lung pathology and improved survival rate in animal models (45, 47). Concurrently, the independence of TMPRSS2 renders the omicron variant a broader spectrum of cellular tropism to infect ACE2^+^ cells which are more abundant in human bronchi than lungs (48). This explains why the omicron variant prefers to accumulate in upper airways over deep lungs, causing alleviated intrinsic severity once patients are infected (49). Our results became consistent with those findings, where nearly half proportion of patients went through asymptomatic manifestations and lung infiltration did not induce severe pathological changes in most patients (e.g., consolidation, crazy paving pattern).

Here our study had limitations. First, our patient number was small. This further made the patient number in different subgroups even smaller. Given the recent escalation of the omicron outbreak and the increasing portion of patients with no symptom or no need for hospitalization, clinical data became less available. Second, there was no severe or deceased patient in our study, so we could not have access to analyze the possible risk factors associated with severity or mortality of COVID-19 infection by the omicron variant. Similarly, our study contained patients with a median age of 33.0 (IQR: 24.0–45.5). Thus, this study might not elucidate much of vaccine effectiveness and viremic effect in the aged population (>60 years old). Third, this study lacked a continuous monitoring of COVID-19 patients during hospitalization and post hospital discharge. This would make more complete research on the long-term outcome of the omicron variant infection to justify its pathogenic feature and consequence.

## Conclusions

In closing, we investigated the baseline characteristics of COVID-19 patients infected by the omicron variant of SARS-CoV-2 together with findings on its reduced clinical severity. Albeit the high mutation in the omicron variant may effectuate its evasion from the neutralizing antibodies, the functional non-neutralizing machinery and the effective cell-mediated immunity constitute the secure frontline defensing against the viral attack of the omicron variant. Simultaneously, the infection route and intrinsic virulence of the omicron variant greatly alter, thereby attenuating its detrimental effect on lungs. Nonetheless, booster jabs can provide the reinforced protection against COVID-19 severity and mortality, especially for those with compromised immune system.

## Data Availability Statement

The raw data supporting the conclusions of this article will be made available by the authors, without undue reservation.

## Ethics Statement

The studies involving human participants were reviewed and approved by Research Ethics Commission of the Fifth People's Hospital of Suzhou (TFPHS, the Affiliated Infectious Diseases Hospital of Soochow University), China. Written informed consent from the participants or their legal guardian/next of kin was not required to participate in this study in accordance with the national legislation and the institutional requirements.

## Author Contributions

JiaZ and ZT conceived the idea and designed the study. JiaZ, NC, DZ, ZH, and ZT contributed to the data processing and table/figure preparation. NC, JinZ, and ZT contributed to the statistical analysis. All authors contributed to the manuscript writing and approved the manuscript submission.

## Conflict of Interest

The authors declare that the research was conducted in the absence of any commercial or financial relationships that could be construed as a potential conflict of interest.

## Publisher's Note

All claims expressed in this article are solely those of the authors and do not necessarily represent those of their affiliated organizations, or those of the publisher, the editors and the reviewers. Any product that may be evaluated in this article, or claim that may be made by its manufacturer, is not guaranteed or endorsed by the publisher.
